# STE029逆转肺腺癌EGFR-TKI耐药及其机制研究

**DOI:** 10.3779/j.issn.1009-3419.2022.102.46

**Published:** 2022-11-20

**Authors:** 麟 黄, 梅 侯, 洁薇 刘, 洋 李, 旺 沈, 清华 周

**Affiliations:** 1 610041 成都，肺癌中心/肺癌研究所，四川大学华西医院 Sichuan Lung Cancer Center, Sichuan Lung Cancer Institute, West China Hospital, Sichuan University, Chengdu 610041, China; 2 300052 天津，天津市肺癌研究所，天津市肺癌转移与肿瘤微环境重点实验，天津医科大学总医院 Tianjin Key Laboratory of Lung Cancer, Metastasis and Tumor Microenvironment, Tianjin Lung Cancer Institute, Tianjin Medical University General Hospital, Tianjin 300052, China

**Keywords:** STE029, EGFR-TKI, 耐药, 细胞凋亡, 细胞周期, 肺腺癌, STE029, EGFR-TKI, Resistance, Apoptosis, Cell cycle, Lung adenocarcinoma

## Abstract

**背景与目的:**

表皮生长因子受体酪氨酸激酶抑制剂（epidermal growth factor receptor-tyrosine kinase inhibitor, EGFR-TKI）获得性耐药和原发性耐药至今仍是临床治疗晚期非小细胞肺癌（non-small cell lung cancer, NSCLC）的瓶颈。STE029是一种同时具有羟甲基戊二酸单酰辅酶A还原酶（3-hydroxy-3-methylglutarylcoenzyme A reductase, HMGCR）抑制剂抗肿瘤作用和肿瘤特异性细胞膜靶向功能的新型抗肿瘤药物。本研究旨在探讨STE029逆转肺腺癌EGFR-TKI的耐药机制。

**方法:**

STE029、吉非替尼单药及联合用药分别处理PC9、PC9/BB4、A549细胞，检测各细胞株的活性变化、细胞增殖、细胞凋亡，鉴定EGFR/PI3K/Akt信号通路、细胞周期及凋亡相关蛋白的表达；同时观察STE029、吉非替尼单药及联合用药对PC9、PC9/BB4裸鼠皮下移植瘤生长的影响。

**结果:**

① PC9细胞为*EGFR*突变的敏感细胞，PC9/BB4细胞为*EGFR*突变的获得性耐药细胞，A549细胞为非*EGFR*突变的耐药细胞；②STE029联合吉非替尼作用于PC9、PC9/BB4、A549细胞后，吉非替尼的半数抑制浓度（50% inhibitory concentration, IC_50_）值较对照组均显著降低（*P* < 0.05）；③STE029联合吉非替尼可抑制PC9、PC9/BB4细胞的增殖（*P* < 0.001），诱导A549细胞凋亡增加（*P* < 0.01）；④在PC9、PC9/BB4细胞中，联合用药组较单药组p-EGFR、p-Akt的表达明显下调（*P* < 0.000,1），GSK-3β表达升高（*P* < 0.001），其下游p-Cyclin D1及Cyclin D1表达明显降低（*P* < 0.001），cleaved caspase-8、caspase-8、cleaved caspase-9、caspase-9的表达在各给药组间未见明显差异（*P* > 0.05）；在A549细胞中，联合用药组p-Akt表达明显下调（*P* < 0.001），cleaved caspase-8、cleaved caspase-9表达均升高（*P* < 0.001），而GSK-3β、p-Cyclin D1、Cyclin D1、caspase-8、caspase-9的蛋白表达在各给药组间则无明显差异（*P* > 0.05）；⑤裸鼠体内，联合用药组PC9皮下移植瘤的生长明显受到抑制，差异有明显统计学意义（*P* < 0.01）；联合用药组PC9/BB4皮下移植瘤的生长速率亦明显降低（*P* < 0.05）。

**结论:**

STE029可在体内外明显增加非*EGFR*-T790M突变耐药的人肺腺癌细胞对吉非替尼的敏感性，其机制可能与STE029通过EGFR/PI3K/Akt信号通路调节GSK-3β、Cyclin D1的表达、阻滞细胞增殖、诱导细胞凋亡有关。

## Abstract (en)

**Background and objective:**

Acquired and primary resistance to epidermal growth factor receptor tyrosine kinase inhibitor (EGFR-TKI) is still the bottleneck of clinical treatment of advanced non-small cell lung cancer (NSCLC). STE029 is a novel anticancer drug which consists of 3-hydroxy-3-methylglutarylcoenzyme A reductase (HMGCR) inhibitor and novel cancer cell membrane targeting molecular. This study aimed to investigate the reversal mechanism of EGFR-TKI resistance by STE029 in lung adenocarcinoma.

**Methods:**

CCK8 test was used to test the cell viability and survival rate of *EGFR* mutated PC9 cell (Gefitinib sensitive), PC9/BB4 cell (acquired Gefitinib resistant), and EGFR wild type A549 cell after treatment of STE029, Gefitinib or combination of both. EdU test was applied to detect changes in cell cycle and Hoechst 33258 was applied to detect apoptosis rate in overcoming the EGFR-TKI resistance. The activity of EGFR/PI3K/Akt, cell cycle and apoptosis signal pathways were examined. *In vivo*, nude mice were exposed to STE029, Gefitinib and STE029+Gefitinib for 5 wk. And the the tumor volume was measured and tumor weight was obtained on the last day.

**Results:**

(1) PC9 cells was highly sensitive to Gefitinib, while PC9/BB4 and A549 cell showed significant resistance to Gefitinib treatment; (2) STE029+Gefitinib treatment could significantly decrease the 50% inhibitory concentrarion (IC_50_) of Gefitinib in PC9, PC9/BB4 and A549 cells (*P* < 0.05, respectively); (3) In PC9 and PC9/BB4 cells, STE029+Gefitinib can block cell cycle and inhibit cell proliferation (*P* < 0.001), while there was no significant difference in apoptosis rate among three drug intervention groups (*P* > 0.05); However, apoptosis rate was increased in STE029+Gefitinib group in A549 cell (*P* < 0.01), while no significance detected in cell proliferation (*P* > 0.05). (4) In PC9 and PC9/BB4 cells, the combination of STE029 and Gefitinib could downregulate p-EGFR, p-Akt, p-Cyclin D1 and Cyclin D1 (*P* < 0.001), and upregulate the expression of GSK-3β (*P* < 0.001), and the expression of cleaved caspase-8, caspase-8 cleaved caspase-9, caspase-9 showed no difference among groups (*P* > 0.05). In A549 cells, the combination of STE029 and Gefitinib could downregulate p-Akt (*P* < 0.001) and upregulate cleaved caspase-8 and cleaved caspase-9 (*P* < 0.001); (5)*In vivo*, the combination of STE029 and Gefitinib effectively inhibited tumor development and progression compared to STE029 alone or Gefitinib alone, with significant difference (*P* < 0.05) in PC9 and PC9/BB4 xenografted tumor.

**Conclusion:**

STE029 could sensitize Gefitinib by inhibiting EGFR/PI3K/Akt pathway, blocking the tumor cell cycle and proliferation and inducing apoptosis through caspase-8 and caspase-9 dependent pathway. STE029 deserves further investigations in overcoming EGFR-TKI resistance in lung cancer.

近20年来，由于传统的放化疗手段对晚期肺癌的疗效有限，特异性差，在提高肺癌的总治愈率及总生存率方面收效甚微^[1]^。为减少传统细胞毒性药物的副反应、提高药物治疗的特异性、改善临床疗效，针对肿瘤的分子靶向药物已得到了广泛的临床应用。

表皮生长因子受体（epidermal growth factor receptor, EGFR）在肺癌的发生、发展中起到关键作用。吉非替尼是第一代EGFR酪氨酸激酶抑制剂（tyrosine kinase inhibitor, TKI）家族中的一员，可通过与ATP或底物竞争结合EGFR的酪氨酸激酶结构域，特异性抑制EGFR酪氨酸激酶活性，从而抑制其下游Ras/Raf/MAPK、ERK1/2、PI3K/Akt/mTOR等多种促进细胞增殖、迁移、黏附、凋亡抑制和血管新生的信号传导通路，进而抑制肿瘤生长和转移^[2]^。然而，几乎所有对吉非替尼敏感的患者在治疗6个月-12个月后均会产生获得性耐药，导致疾病进展^[3]^。因此，防止或逆转非小细胞肺癌（non-small cell lung cancer, NSCLC）细胞对EGFR-TKI的获得性耐药，逐渐成为了近年来肺癌研究的热点和前沿课题。作为第三代EGFR-TKI的代表药物，AZD9291有效克服了部分NSCLC患者在接受第一代EGFR-TKI治疗后的获得性耐药，它的药物靶点为*EGFR* T790M抗药性突变，其疗效和安全性已得到广泛认可^[4-6]^。然而，目前已有研究^[4]^发现，在接受AZD9291治疗约9个月后，多数患者会再次耐药，对此尚无确定有效的治疗方案；并且，TKI获得性耐药的*EGFR*突变患者中仅有50%存在T790M耐药突变^[7, 8]^，仍有近半数的TKI获得性耐药患者无法从AZD9291的治疗中获益。因此，进一步研究和开发针对EGFR-TKI获得性耐药尤其是非T790M突变的获得性耐药的药物和治疗方法具有重要的意义。

STE029是由羟甲基戊二酸单酰辅酶A还原酶（3-hydroxy-3-methylglutarylcoenzyme A reductase, HMGCR）抑制剂与一种肿瘤细胞细胞膜特异性靶向分子耦合而成，具有高度亲脂性，其分子式为：C_65_H_85_C_1_N_2_O_9_S。HMGCR抑制剂的代表性药物有辛伐他汀、洛伐他汀等，均以其强效降低胆固醇的作用而被公众所熟知。越来越多的临床和基础研究^[9-13]^表明，HMGCR抑制剂能抑制肿瘤的发生发展，其抗肿瘤作用的机制主要包括以下几个方面^[14-16]^：抑制增殖和促进凋亡、抗血管生成作用、抑制肿瘤干细胞以及化疗协同作用。除抗肿瘤作用之外，近年来已有学者开始研究HMGCR抑制剂在逆转肺癌耐药中的作用。一项II期临床研究结果^[17]^显示，在EGFR野生型的非腺癌NSCLC患者中，辛伐他汀与吉非替尼联合组的客观缓解率（40% *vs* 0%, *P*=0.043）及中位无进展生存期（3.6个月*vs* 1.7个月，*P*=0.027）均明显优于吉非替尼单药组。Hwang及其团队^[18]^则发现，辛伐他汀可通过抑制Akt/β-catenin信号传导途径，下调Survivin表达，诱导凋亡，最终增加*EGFR* T790M突变的NSCLC细胞对吉非替尼的敏感性。STE029的另一部分为具有肿瘤细胞特异性的细胞膜靶向分子，我团队前期研究表明其对肺腺癌非T790M突变的EGFR-TKI耐药细胞具有抗肿瘤作用，与HMGCR抑制剂耦合后，在理论上可提高HMGCR抑制剂对肿瘤细胞的特异性杀伤作用。

因此，为了解决NSCLC中非T790M突变的EGFR-TKI耐药的难题，本研究旨在探究STE029克服NSCLC吉非替尼耐药的作用机制，为NSCLC治疗中EGFR-TKI耐药问题的解决提供新的思路。

## 1 材料与方法

### 1.1 细胞和主要试剂

人肺腺癌*EGFR*突变阳性吉非替尼敏感PC9细胞株、人肺腺癌*EGFR*突变阳性吉非替尼耐药PC9/BB4细胞株、人肺腺癌EGFR野生型A549细胞株均由天津市肺癌研究所提供；吉非替尼（ZD1839）购自美国Selleck Chemicals公司；STE029由美国大仁公司惠赠；CCK8试剂购于日本同仁化学研究所；EdU细胞增殖检测试剂盒购自广州锐博生物科技公司；Hoechst 33258细胞凋亡染色试剂盒购自上海碧云天生物技术研究所；EGFR兔单抗、p-EGFR兔单抗、Akt兔单抗、p-Akt兔单抗、Cyclin D1兔单抗、p-Cyclin D1兔单抗、caspase-8鼠单抗、cleaved caspase-8兔单抗、caspase-9兔单抗、cleaved caspase-9兔单抗、Gsk-3β兔单抗、β-actin鼠单抗均购自美国Cell Signaling Technology公司；辣根酶标记山羊抗兔IgG二抗、辣根酶标记山羊抗小鼠IgG二抗均购自上海碧云天生物技术研究所。

### 1.2 实验分组

吉非替尼和STE029分别用DMSO和无水乙醇溶解，体外实验中不同药物处理分组如下（CCK8实验及体内实验单独说明）：对照组、STE029单药组、吉非替尼单药组、STE029+吉非替尼联合用药组；PC9细胞的给药浓度为STE029 1 μmol/L、吉非替尼0.05 μmol/L；PC9/BB4、A549细胞的给药浓度为STE029 6 μmol/L、吉非替尼1 μmol/L；对照组予完全培养基正常培养。

### 1.3 CCK-8法检测细胞增殖情况

收集进入对数生长期、生长状态良好的细胞，PBS漂洗，胰酶消化，完全培养基重悬。取96孔板，每孔加入100 μL细胞悬液铺板，同时设置调零孔（仅含100 μL完全培养基）。接种24 h后细胞贴壁，吸出残余培养基，加入吉非替尼终浓度分别为0 μmol/L、0.003,9 μmol/L、0.015,6 μmol/L、0.062,5 μmol/L、0.25 μmol/L、1 μmol/L、4 μmol/L、8 μmol/L、16 μmol/L、32 μmol/L的完全培养基100 μL；PC9细胞加入STE029终浓度分别为0 μmol/L、0.5 μmol/L、1 μmol/L、2 μmol/L的完全培养基100 μL，PC9/BB4及A549细胞加入STE029终浓度分别为1 μmol/L、2 μmol/L、4 μmol/L的完全培养基100 μL，分别置于37 ℃、5%CO_2_孵箱中培养。于加含药培养基72 h后每孔加入含10 μL CCK-8的DMEM培养基（无血清）100 μL，将培养板在培养箱内孵育2 h。用酶标仪测定各孔在450 nm处的吸光值。计算细胞抑制率，应用曲线回归分析，求出吉非替尼、STE029半数抑制浓度（50% inhibitory concentration, IC_50_）。以上实验均重复3次。

### 1.4 EdU检测细胞增殖

取对数生长细胞，以每孔5×10^3^个/mL细胞接种于96孔板中，培养24 h后加药，继续培养48 h后检测细胞增殖。用细胞完全培养基按1,000:1的比例稀释EdU溶液，每孔加入100 µL 50 µmol/L EdU培养基孵育2 h，弃培养基，PBS清洗细胞1次-2次，每次5 min；每孔加入50 µL细胞固定液（含4%多聚甲醛的PBS）室温孵育30 min，弃固定液；每孔加入50 µL 2 mg/mL甘氨酸，脱色摆床孵育5 min后每孔加入100 µL PBS清洗5 min，每孔加入100 µL渗透剂（含0.5%Triton X-100的PBS）脱色摇床孵育10 min；PBS清洗5 min，每孔加入100 µL的1×Apollo^®^染色反应液（按说明书），避光、室温、脱色摇床孵育30 min，加入100 µL渗透剂脱色摆床清洗3次，10 min/次；每孔加入100 µL去离子水稀释后的Hoechest 33342染液（100:1），避光、室温、脱色摇床孵育30 min，PBS清洗3次，加入100 µL PBS上机检测。以上实验均重复3次。

### 1.5 Hoechst 33258检测细胞凋亡

取洁净盖玻片在70%乙醇中浸泡5 min或更长时间，无菌超净台内吹干或用无菌的PBS或0.9%NaCl等溶液洗涤3遍，再用细胞培养液洗涤1遍。将盖玻片置于6孔板内，种入细胞培养过夜。刺激细胞发生凋亡后，吸尽培养液，加入0.5 mL固定液，固定10 min。去固定液，用PBS或0.9%NaCl洗2遍，每次3 min，吸尽液体。洗涤时宜用摇床或手动晃动。加入0.5 mL Hoechst 33258染色液，染色5 min，摇床或手动晃动数次。去染色液，用PBS或0.9%NaCl洗2遍，每次3 min，吸尽液体。洗涤时宜用摇床或手动晃动。滴1滴抗荧光淬灭封片液于载玻片上，盖上贴有细胞的盖玻片，让细胞接触封片液。荧光显微镜可检测到呈亮蓝色的细胞核。以上实验均重复3次。

### 1.6 Western blot检测EGFR/Akt/GSK-3β/Cyclin D1、Caspase蛋白表达

药物处理48 h后，收集细胞，加入适量蛋白裂解液，充分混匀，冰上孵育30 min，4 ℃、13,000 rpm离心15 min，取上清，-80 ℃保存。配制SDS-PAGE凝胶，按标准方法进行电泳、转膜、封闭，目的蛋白抗体4 ℃孵育过夜，二抗常温孵育2 h，常规暗室ECL显影，分析图片。以上实验均重复3次。

### 1.7 体内实验探讨STE029逆转肺腺癌吉非替尼耐药的作用

实验动物为4周龄-6周龄雌性BALB/c-Nude品系小鼠（胸腺细胞免疫缺陷鼠），实验和饲养条件按照SPF动物的规范要求。建立PC9、PC9/BB4裸鼠皮下移植瘤模型：取生长状态良好的细胞，每只BALB/c-Nude裸鼠接种100 μL（6×10^7^个/mL）于右侧腹股沟处，每种细胞共接种20只，随机分为4组，每组5只，并进行编号。待肿瘤体积达100 mm^3^以上时开始给药，剂量如下：吉非替尼单药10 mg/kg；STE029单药8 mg/kg；联合用药组予吉非替尼10 mg/kg+STE029 8 mg/kg；对照组予含2.5%无水乙醇、1%Tween80的无菌三蒸水100 μL；上述药物均腹腔注射给药，每周2次。共给药5周，每周测量2次肿瘤体积、裸鼠体重及肿瘤生物发光强度。脱颈法处死裸鼠后，无菌条件下小心剥离种植瘤，瘤体称重，游标卡尺测量瘤体的体积。计算抑瘤率。

### 1.8 统计学方法

应用SPSS 22.0统计软件及GraphPad Prism 6.0进行统计学分析及图像处理。应用曲线回归分析求IC_50_。移植瘤生长趋势的比较采用重复测量方差分析。多组间比较采用方差分析，两组间比较采用*t*检验。界值（双侧0.05为检验标准），*P* < 0.05为差异有统计学意义。

## 2 结果

### 2.1 STE029联合吉非替尼对PC9、PC9/BB4、A549细胞体外抑制率的检测

应用曲线回归分析分别计算吉非替尼、STE029在各个细胞株中的IC_50_（表 1）。CCK8法检测结果显示，吉非替尼与STE029联合处理72 h与吉非替尼单药处理72 h的各细胞株相比（表 2）：①吉非替尼敏感的PC9细胞的吉非替尼IC_50_显著下降，差异有统计学意义（*P* < 0.05）；②吉非替尼耐药的PC9/BB4细胞的吉非替尼IC_50_显著下降，差异有统计学意义（*P* < 0.05）；③EGFR野生型的A549细胞的吉非替尼IC_50_在STE029浓度为8 μmol/L处理后显著下降，差异有统计学意义（*P* < 0.05）。分别绘制不同药物处理下各细胞株的药物浓度-存活率曲线，以药物浓度（μmol/L）为横轴，以存活率（%）为纵轴作图（图 1）。

**表 1:** 人肺腺癌细胞株PC9、PC9/BB4、A549细胞株对吉非替尼、STE029的IC_50_ Comparison of Gefitinib IC_50_ and STE029 IC_50_ in PC9, PC9/BB4, A549 cell lines

Index	IC_50_ (*μ*mol/L) (Mean, 95%CI)			*P*
	PC9	PC9/BB4	A549	
Gefitinib	0.12 (0.07-0.21)	11.41 (10.67-12.20)	14.39 (13.32-15.55)	0.001^a^
				< 0.000,1^b^
				0.054^c^
STE029	2.48 (2.17-2.85)	7.80 (5.92-10.26)	8.26 (7.64-8.92)	0.005^a^
				0.007^b^
				0.320^c^
^a^: comparison between PC9 cell lines and PC9/BB4 cell lines; ^b^: comparison between PC9 cell lines and A549 cell lines; ^c^: comparison between PC9/BB4 cell lines and A549 cell lines. IC_50_: 50% inhibitory concentration.				

**表 2:** 人肺腺癌细胞株PC9、PC9/BB4、A549细胞株联合不同浓度STE029处理后对吉非替尼IC_50_的影响 Comparison of Gefitinib IC_50_ in PC9, PC9/BB4, A549 cell lines after in combination with STE029

Cell line	STE029 concentration	Gefitinib IC_50_ (*μ*mol/L) (Mean, 95%CI)	*P* ^a^
PC9	0 *μ*mol/L	0.128 (0.075-0.218)	-
	0.5 *μ*mol/L	0.006 (0.003-0.009)	0.007,4
	1 *μ*mol/L	0.004 (0.002-0.008)	0.013,3
	2 *μ*mol/L	0.003 (0.002-0.006)	0.011
PC9/BB4	0 *μ*mol/L	11.410 (10.670-12.200)	-
	4 *μ*mol/L	2.606 (1.988-3.417)	0.021
	6 *μ*mol/L	1.910 (1.402-2.603)	0.031
	8 *μ*mol/L	0.924 (0.580-1.472)	0.034
A549	0 *μ*mol/L	14.390 (13.320-15.550)	-
	4 *μ*mol/L	10.760 (8.344-13.870)	0.135
	6 *μ*mol/L	10.660 (8.337-13.640)	0.098
	8 *μ*mol/L	6.251 (4.934-7.919)	0.004
^a^: comparison of Gefitinib IC_50_ under different concentration of STE029 in PC9, PC9/BB4 and A549 cell lines, respectively.			

**图 1:**
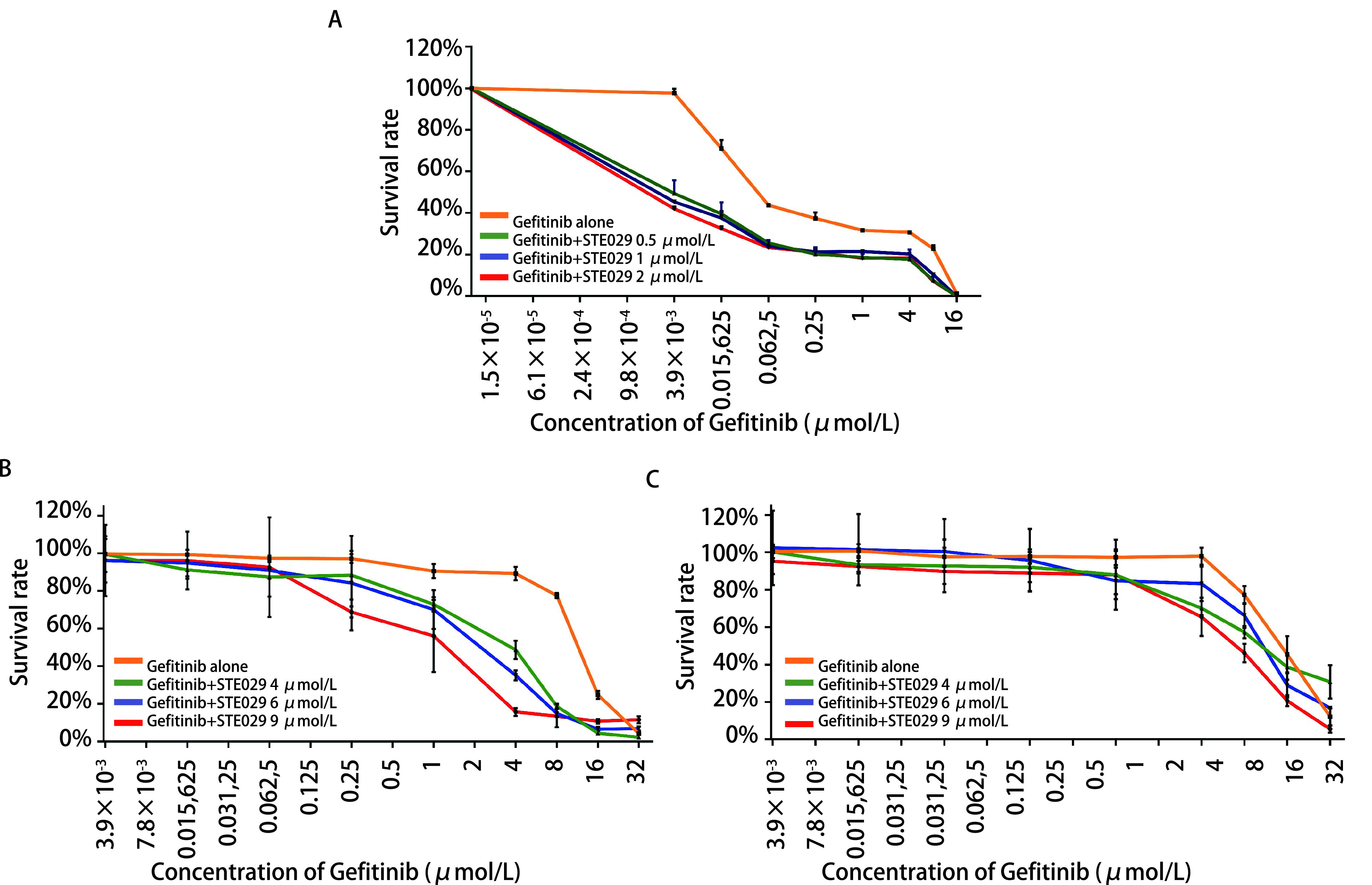
各细胞株在不同药物处理方式下的存活率回归曲线图。A：PC9细胞；B：PC9/BB4细胞；C：A549细胞。 Regression curve of survival rate treated with different combination of Gefitinib and STE029. A: PC9 cell; B: PC9/BB4 cell; C: A549 cell.

### 2.2 吉非替尼、STE029单药及联合用药对PC9、PC9/BB4、A549细胞增殖及凋亡的影响

经药物处理后，在PC9及PC9/BB4细胞中，与对照组相比，药物处理组处于增殖状态的细胞较少（各组之间无明显统计学差异），而联合用药组较单药组处于增殖状态的细胞明显减少（*P* < 0.001）；在A549细胞中，药物处理组较对照组处于增殖状态的细胞少（*P* < 0.001），而联合用药组与单药组之间增殖细胞比例无明显差异（图 2）；经Hoechst 33258染料染色后，对照组细胞生长旺盛，呈淡蓝色；在A549细胞中，联合用药组细胞的细胞核固缩、碎裂，大部分细胞发生了凋亡，呈高亮蓝色（与对照组相比，*P* < 0.01）；在PC9及PC9/BB4细胞中，各药物处理组细胞均可见凋亡细胞，但各组之间无明显差异（*P* > 0.05）（图 3）。

**图 2:**
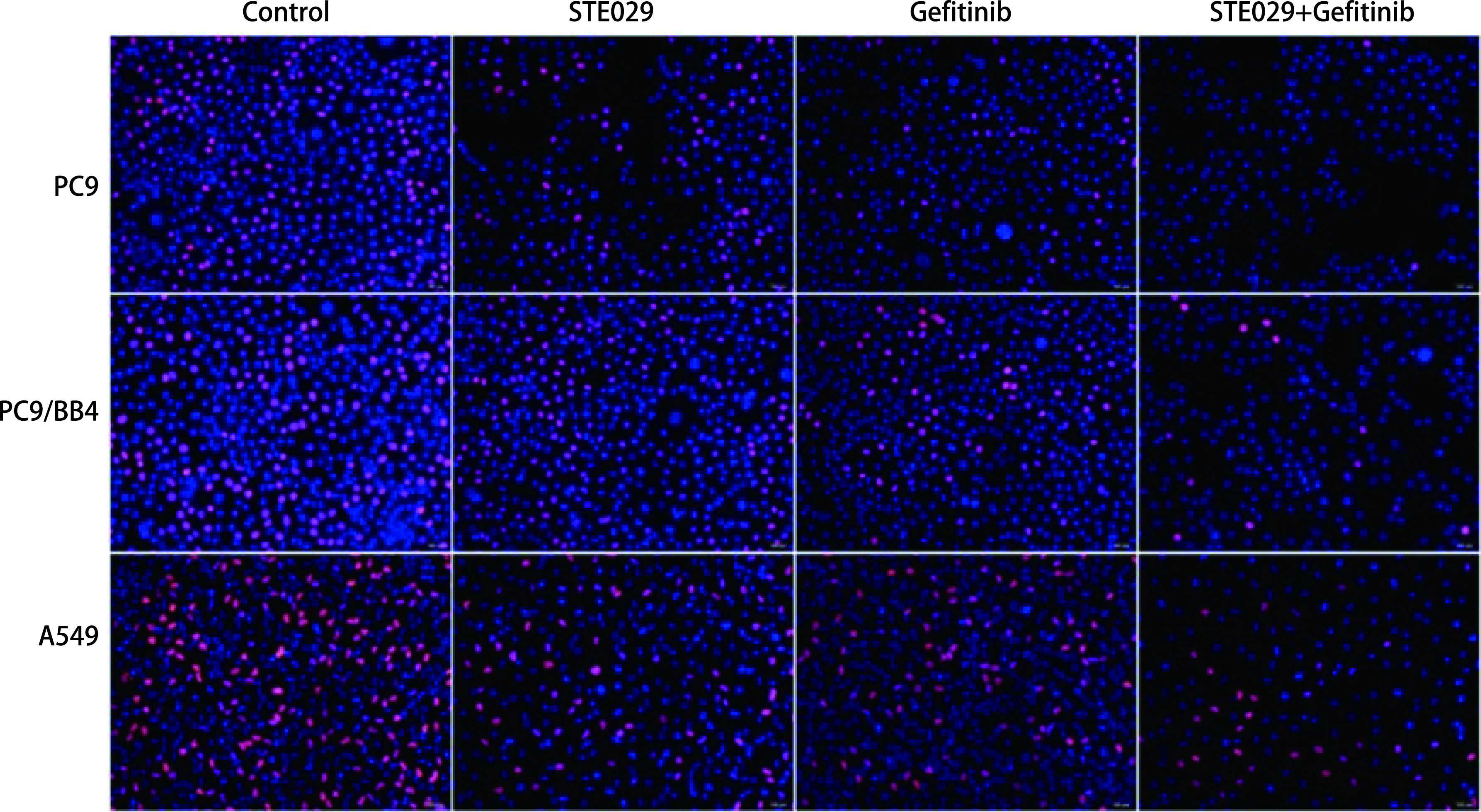
PC9、PC9/BB4、A549细胞在不同药物处理后检测细胞增殖状态。EdU法检测细胞增殖（×200），处于增殖状态的细胞为红色。 Cell proliferation was tested by EdU fluorescent staining in PC9, PC9/BB4, A549 cells after treated with different combination of Gefitinib and STE029. Proliferated cells showed red signal by EdU staining (×200).

**图 3:**
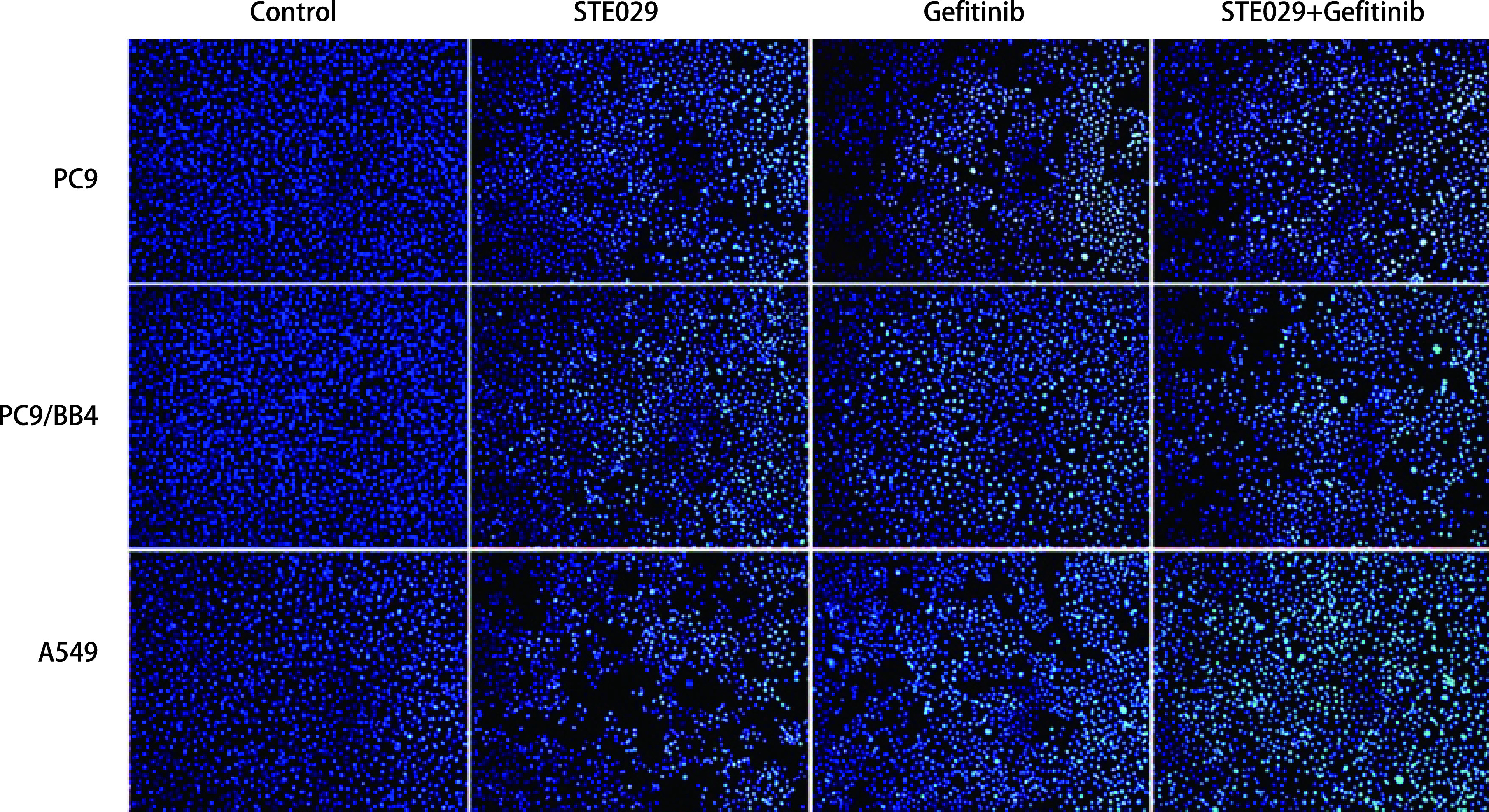
PC9、PC9/BB4、A549细胞在不同药物处理后检测细胞凋亡状态。Hoechst 33258染液染细胞后，凋亡细胞核固缩，呈亮蓝色（×200）。 Cell proliferation was tested by EdU fluorescent staining in PC9, PC9/BB4, A549 cells after treated with different combination of Gefitinib and STE029. Cell apoptosis was tested by Hoechst 33258 fluorescent staining, and apoptosis cells showed highlight signal (×200).

### 2.3 STE029联合吉非替尼对PC9、PC9/BB4及A549细胞EGFR通路、细胞周期、凋亡相关蛋白表达水平的影响

在人肺腺癌PC9、PC9/BB4、A549细胞中，EGFR、Akt、GSK-3β及β-actin的蛋白表达量未见明显差异。在PC9及PC9/BB4细胞中，与各对应的单药组相比，联合用药组p-EGFR、p-Akt蛋白的表达明显下调（*P* < 0.000,1），GSK-3β的蛋白表达量显著升高（*P* < 0.001），其下游p-Cyclin D1及Cyclin D1的蛋白表达量显著降低（*P* < 0.001）；在A549细胞中，与各单药组相比，联合用药能显著抑制p-Akt的表达（*P* < 0.001），而GSK-3β及p-Cyclin D1、Cyclin D1的蛋白表达却未见明显差异（图 4A）。在人肺腺癌PC9、PC9/BB4、A549细胞中，caspase-9、caspase-9及β-actin的蛋白表达量未见明显差异。与各对照组相比，STE029单药、吉非替尼单药及二者联合用药均能诱导凋亡发生，cleaved caspase-8及cleaved caspase-9的蛋白表达量均有上调（*P* < 0.05）：在PC9、PC9/BB4细胞株，与单药组相比，联合用药组cleaved caspase-8及cleaved caspase-9的蛋白表达量未见显著升高；在A549细胞中，联合用药组cleaved caspase-8及cleaved caspase-9的蛋白较各单药组均明显上调（*P* < 0.001）（图 4B）。

**图 4:**
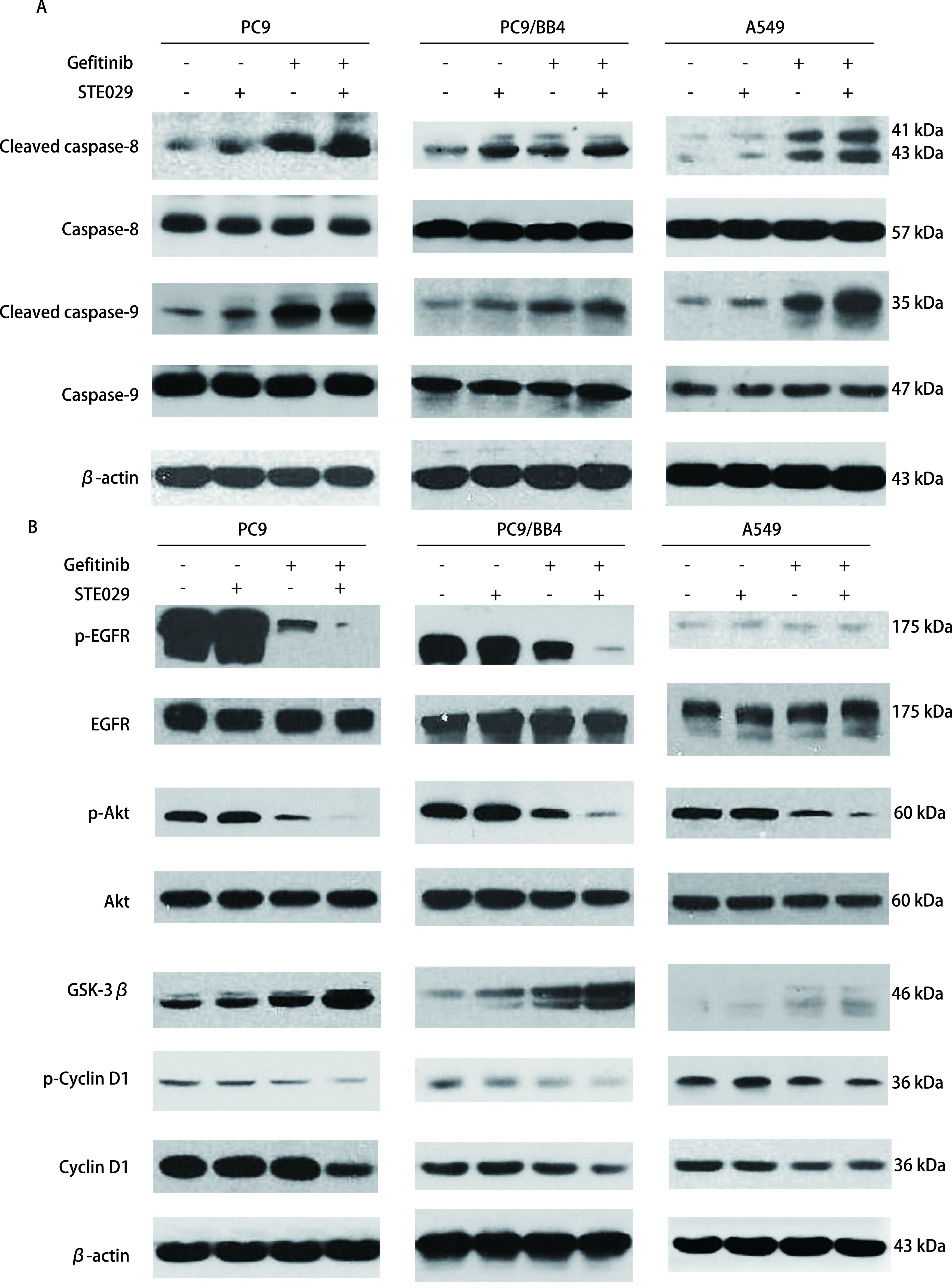
吉非替尼、STE029联合应用对PC9、PC9/BB4、A549细胞EGFR通路及细胞周期（A）、细胞凋亡相关蛋白（B）表达水平的影响 Western-blotting for EGFR signaling pathway and cell cycle-related proteins (A) and cell apoptosis-related proteins (B) in PC9, PC9/BB4, A549 cell lines influenced by Gefitinib or STE029 or Gefitinib+STE029. EGFR: epidermal growth factor receptor.

### 2.4 吉非替尼、STE029单药及联合用药对PC9、PC9/BB4移植瘤成瘤性的影响

通过观察到皮下移植瘤生长速度发现，用同等浓度的吉非替尼处理相同时间后，PC9皮下移植瘤生长速度明显慢于PC9/BB4皮下移植瘤，二者间差异有明显统计学意义（*P* < 0.000,1）（图 5A），由此可见，与PC9相比，PC9/BB4移植瘤对吉非替尼显著耐药。

**图 5:**
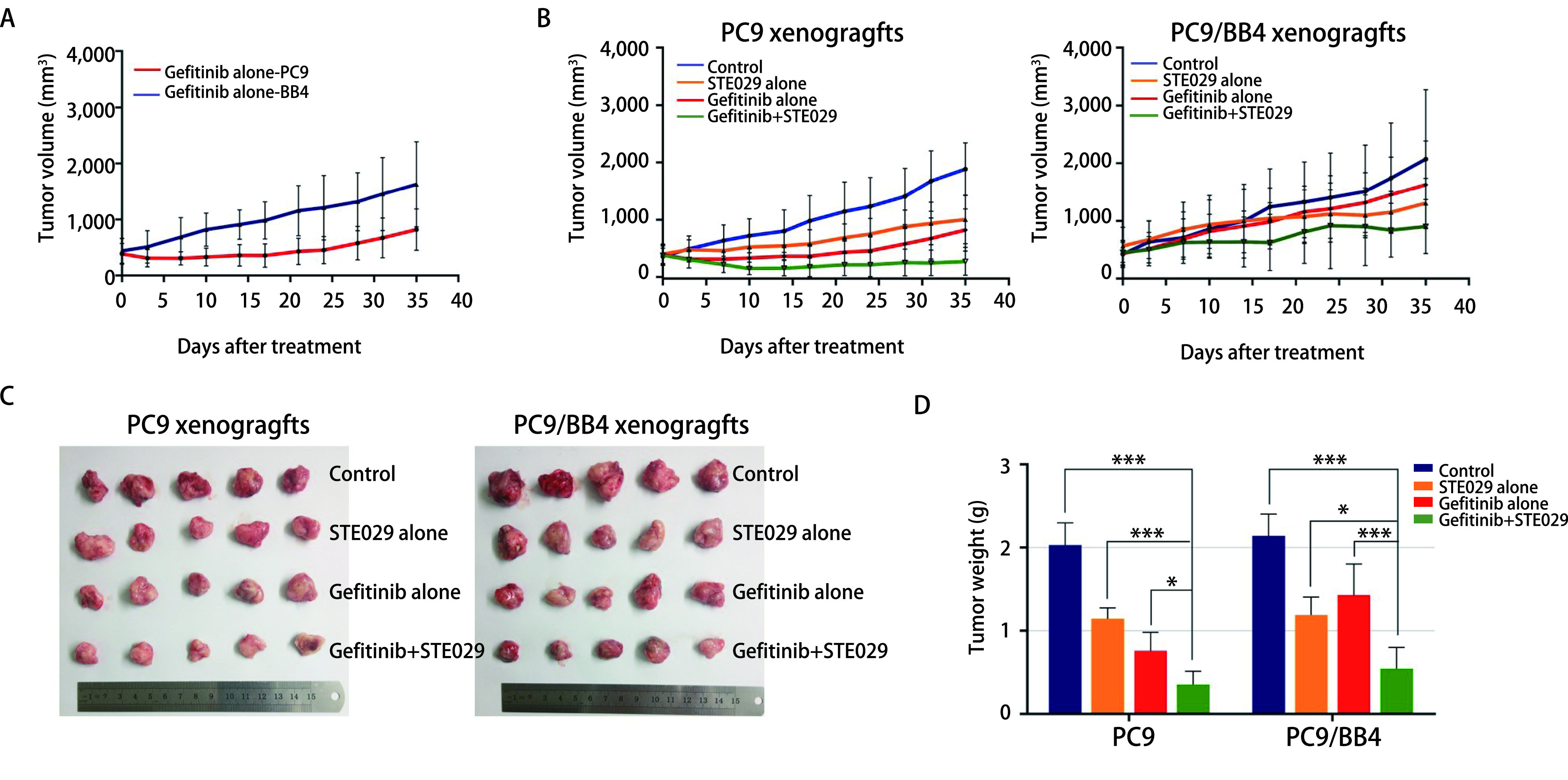
STE029联合吉非替尼对裸鼠体内皮下移植瘤生长的影响。A：同等剂量吉非替尼干预后，BALB/c-Nude裸鼠体内PC9、PC9/BB4皮下原发移植瘤的生长趋势，PC9/BB4皮下原发移植瘤较PC9对吉非替尼显著耐药（*P* < 0.000,1）；B：药物干预后，BALB/c-Nude裸鼠体内PC9、PC9/BB4皮下原发移植瘤的生长趋势；C：对照组、STE029单药组、吉非替尼单药处理组和联合用药组在BALB/c-Nude裸鼠体内皮下形成的原发移植瘤；D：不同处理组皮下移植瘤第35天时瘤体重量。 STE029 overcomes Gefitinib resistance *in vivo*. Compared to PC9 xenografts, PC9/BB4 xenografts showed significant resistance to Gefitinib (*P* < 0.000,1) (A); Tumor growth curves (B), Macroscopic appearance (C) and tumor weight (D) of xenografts harvested on day 35 for PC9, PC9/BB4 xenografts according to different treatments. ***: *P* < 0.001; *: *P* < 0.05.

经重复测量的方差分析显示，PC9皮下移植瘤中各用药组较对照组皮下移植瘤的生长速度有明显差异（*P* < 0.01）；STE029单药与吉非替尼单药组之间肿瘤生长速度亦有明显差异（*P* < 0.000,1）；与STE029单药组及吉非替尼单药组相比，联合用药组皮下移植瘤的生长明显受到抑制，移植瘤体积间差异均有明显统计学意义（联合用药*vs* STE029单药：*P*=0.000,4；联合用药*vs*吉非替尼单药：*P*=0.005,4）（图 5B）；PC9/BB4皮下移植瘤中STE029单药组皮下移植瘤生长速度与对照组及吉非替尼单药组相比无明显差异（STE029单药组*vs*对照组：*P*=0.226,1；STE029单药组*vs*吉非替尼单药组：*P*=0.903,8）；吉非替尼单药组及联合用药组与对照组相比，移植瘤生长缓慢（吉非替尼单药*vs*对照组：*P*=0.010,4；联合用药组*vs*对照组：*P*=0.007,9）；联合用药组皮下移植瘤生长速度显著低于单药组（联合用药组*vs* STE029单药：*P* < 0.000,1；联合用药组*vs*吉非替尼单药：*P*=0.008,5）（图 5B）。

连续用药5周后脱颈法处死裸鼠，取出皮下移植瘤，如图 5C所示，各用药组之间裸鼠皮下移植瘤大小有明显差异。经*F*检验，各组间皮下移植瘤的重量均存在明显差异（*P* < 0.000,1）。STE029单药、吉非替尼单药及联合用药组的皮下移植瘤的重量均明显低于对照组（*P* < 0.01）；与STE029单药或吉非替尼单药组相比，联合用药组皮下移植瘤的重量显著降低，差异有统计学意义（*P* < 0.05），具体结果见表 3及图 5D。

**表 3:** PC9、PC9/BB4皮下移植瘤第35天时瘤体重量（g） Tumor weight evaluated on day 35^th^ according to different treatments

Cell line	Group	*n*	Tumor weight (g)	*F*	*P*
PC9	Control	5	2.030±0.265	63.66	< 0.000,1
	STE029	5	1.144±0.130		
	Gefitinib	5	0.760±0.221		
	STE029+Gefitinib	5	0.350±0.160		
PC9/BB4	Control	5	2.142±0.259	26.99	< 0.000,1
	STE029	5	1.188±0.217		
	Gefitinib	5	1.426±0.378		
	STE029+Gefitinib	5	0.542±0.258		

## 3 讨论

吉非替尼作为第一代EGFR-TKI的代表药物，在*EGFR*敏感突变患者的治疗中能取得较为满意的临床疗效。然而，EGFR-TKI获得性耐药常常在治疗数月后出现，具有EGFR-TKI获得性耐药的患者则无法继续从吉非替尼治疗中获益。吉非替尼也被用于EGFR野生型患者的治疗，往往与含铂双药化疗联合使用，但治疗效果始终差强人意。因此，增加EGFR-TKI在耐药患者中的敏感性是当下基础与临床研究中亟待解决的难题。

前期实验已验证本实验所采用的人肺腺癌PC9、PC9/BB4细胞中*EGFR*基因的外显子19均存在15 bp的缺失突变，属于最为常见的*EGFR* I类突变。并且，PC9/BB4细胞未检测到T790M的突变，说明其获得性耐药的产生与T790M耐药突变之间无明显相关性。本研究采用的人肺腺癌A549细胞为实验室长期稳定培养的EGFR野生型细胞，对吉非替尼天然耐药，亦是研究EGFR-TKI对肺腺癌作用机制较为常用的细胞株。因此，以敏感株PC9细胞为参照，使用PC9/BB4以及A549细胞来研究如何克服NSCLC EGFR-TKI耐药，可为对第三代TKI药物不敏感的患者提供新的治疗思路，具有更为重要的临床意义。

通过体内外实验，本研究证实了STE029可逆转肺腺癌吉非替尼耐药。在体外，STE029抑制肺腺癌细胞增殖，并可显著增加人肺腺癌*EGFR*突变型和野生型细胞对吉非替尼的敏感性；ST029、吉非替尼均可抑制肺腺癌细胞增殖，诱导细胞凋亡，二者联用均可使上述抗肿瘤作用增强。在体内，STE029联合吉非替尼可显著抑制人肺腺癌皮下移植瘤的生长和成瘤。

作为EGFR下游信号通路中最重要的一部分，PI3K/Akt信号传导途径在*EGFR*突变型及野生型细胞耐药及耐药逆转中均发挥了重要作用。已有研究^[19]^表明，PI3K/Akt信号通路在多类肿瘤细胞中呈过度激活状态，能促进肿瘤细胞增殖、迁移、侵袭、血管生成，抑制细胞凋亡以及促进细胞免疫逃逸及耐药性产生。PI3K/Akt信号通路参与肿瘤耐药的机制十分复杂，其中，抗凋亡和细胞周期的激活是其主要发挥作用的两个方面。过度激活的PI3K/Akt可抑制caspase家族成员的活化而抑制凋亡^[20]^。caspase家族在细胞凋亡过程中起到非常重要的作用，其成员分为始动caspase（caspase-1、-2、-4、-5、-8、-9）和效应caspase（caspase-3、-6、-7、-14）两类。一般情况下，当细胞接收到死亡信号后，线粒体释放的Cty C结合并激活凋亡酶激活因子-1（apoptotic protease activating factor-1, Apaf-1），活化的Apaf-1可直接与caspase-9结合，启动细胞凋亡。然而，过度活化的Akt可通过磷酸化caspase-9而阻碍其与上游蛋白的结合，导致凋亡过程无法顺利启动；caspase-8则可被活化的Fas和TNFR所激活，进而启动凋亡过程^[20, 21]^，Akt可通过直接抑制Fas的激活从而抑制caspase-8依赖的细胞凋亡。吉非替尼可通过直接抑制EGFR的激活而抑制其下游Akt的激活已得到广泛证实，亦有研究^[22]^发现HMGCR可抑制内皮细胞中Akt的磷酸化。本研究发现，STE029协同吉非替尼作用于人肺腺癌TKI敏感及耐药细胞，均能显著抑制p-Akt的表达，在*EGFR*基因野生型的A549细胞中还伴随有cleaved caspase-8、cleaved caspase-9蛋白表达的上调，说明STE029克服A549细胞吉非替尼耐药与PI3K/Akt信号通路的抑制以及凋亡的激活有关，但二者之间是否存在直接的调控关系还需进一步实验证实。

GSK-3是一种丝氨酸/苏氨酸蛋白激酶，是一种常见的糖原合成酶激酶的限速酶，有α、β两个亚型。近年来，已有大量的研究^[23-25]^证实，GSK-3β在多种肿瘤中存在异常表达，作为细胞外因子/胞质蛋白（Wnt/β-catenin）信号通路中的重要调节蛋白。GSK-3β是一种负性调节因子，其过表达将通过促进β-catenin的磷酸化进而促进β-catenin的降解，解除β-catenin对核内转录因子TCF/LEF的活化作用，进而下调TCF/LEF目的基因如*c-myc*和*Cyclin D1*的表达，最终达到抑制肿瘤细胞生长的作用^[26, 27]^。Cyclin D1是细胞周期中推动细胞周期由G_1_期进入S期的关键蛋白，抑制Cyclin D1则可将细胞阻滞于G_0_期/G_1_期，无法继续增殖。GSK-3β确切的调控机制目前尚不清楚，有研究^[26]^显示，过度激活的PI3K/Akt信号通路可通过磷酸化GSK-3β而抑制其活性，从而促进原癌基因的表达，促进肿瘤增殖。本研究发现，STE029协同吉非替尼作用于人肺腺癌*EGFR*突变细胞，在抑制磷酸化Akt激活的同时，GSK-3β活性成分在细胞中累积增多，同时伴有Cyclin D1蛋白表达量的显著下调，这说明，STE029克服PC9/BB4细胞吉非替尼耐药与PI3K/Akt信号通路的抑制、GSK-3β高活性及细胞增殖的阻滞有关，但三者之间是否存在直接的调控关系还无法就此定论。

综上，本研究证实了STE029可逆转人肺腺癌*EGFR*突变和野生型细胞对吉非替尼的耐药性。STE029与吉非替尼联合可上调GSK-3β的表达，抑制Cyclin D1的表达，抑制PC9、PC9/BB4细胞的增殖，亦可通过caspase-8、caspase-9途径诱导A549细胞凋亡。然而，不论何种抗肿瘤药物联合应用，在提高疗效的同时也将增加药物毒副作用。因此，如何在提高治疗效果的同时减轻药物不良反应也是后续研究和应用中需要关注和解决的重点问题。
